# Modulation of NK cell activation by exogenous calcium from alginate dressings *in vitro*

**DOI:** 10.3389/fimmu.2023.1141047

**Published:** 2023-04-06

**Authors:** Yara Adib, Maxime Boy, Kevin Serror, Nicolas Dulphy, Céline des Courtils, Laura Duciel, David Boccara, Maurice Mimoun, Marina Samardzic, Martine Bagot, Armand Bensussan, Laurence Michel

**Affiliations:** ^1^ Skin Research Center, Institut National de la Santé et de la Recherche Médicale (INSERM) Unités Mixtes de Recherche (UMRS)_976, Hôpital Saint-Louis, Paris, France; ^2^ Université Paris Cité, Paris, France; ^3^ Laboratoires Brothier, Nanterre, France; ^4^ Department of Immunology, Institut National de la Santé et de la Recherche Médicale (INSERM) Unités Mixtes de Recherche (UMRS)_1160, Hôpital Saint-Louis, Paris, France; ^5^ Service de Chirurgie plastique, reconstructive et esthétique, Hôpital Saint-Louis, Paris, France; ^6^ Service d’Immunologie et Histocompatibilité, Assistance publique-Hôpitaux de Paris (APHP), Hôpital Saint Louis, Paris, France; ^7^ Service de Dermatologie, Assistance publique-Hôpitaux de Paris, Hôpital Saint Louis, Paris, France

**Keywords:** natural killer cells, wound healing, calcium alginate wound dressings, calcium ions, NK cell cytotoxicity

## Abstract

Natural Killer (NK) cells participate in the defense against infection by killing pathogens and infected cells and secreting immuno-modulatory cytokines. Defects in NK cell activity have been reported in obese, diabetic, and elderly patients that are at high risk of developing infected chronic wounds. Calcium alginate dressings are indicated for the debridement during the inflammatory phase of healing. Since calcium ions are major activators of NK cells, we hypothesized that these dressings could enhance NK functions, as investigated *in vitro* herein. Primary human blood NK cells were freshly-isolated from healthy volunteers and exposed to conditioned media (CM) from two alginate dressings, Algosteril^®^ (ALG, pure Ca^2+^ alginate) and Biatain^®^ Alginate (BIA, Ca^2+^ alginate with CMC), in comparison with an exogenous 3mM calcium solution. Our results demonstrated that exogenous calcium and ALG-CM, but not BIA-CM, induced NK cell activation and enhanced their capacity to kill their targets as a result of increased degranulation. NK cell stimulation by ALG depended on the influx of extracellular Ca^2+^ *via* the SOCE Ca^2+^ permeable plasma membrane channels. ALG-CM also activated NK cell cytokine production of IFN-γ and TNF-α through a partly Ca^2+^-independent mechanism. This work highlights the non-equivalence between alginate dressings for NK cell stimulation and shows that the pure calcium alginate dressing Algosteril^®^ enhances NK cell cytotoxic and immuno-modulatory activities. Altogether, these results underline a specific property of this medical device in innate defense that is key for the cutaneous wound healing process.

## Introduction

Natural Killer (NK) cells are innate immune cells forming the first line of defense against infection and malignancy. NK cells are subdivided into two populations, based on CD56 and CD16 expression, which are functionally different. The CD56^dim^ CD16^high^ NK cells constitute the majority of blood NK cells: they are cytotoxic NK cells that directly kill their targets (infectious agents, bacteria/virus-infected cells, or tumoral cells). The CD56^bright^ CD16^low^ ones are regulatory NK cells, secreting immunomodulatory cytokines (1–4).

A large number of structurally distinct receptors, which allow the recognition of their targets, have been characterized on cytotoxic NK cells [*e.g.* activating NKG2D, NKp30, NKp46, DNAM-1, Toll-like receptors TLRs and inhibitory KIRs, NKG2A, ILT2, KLRG1, CD95 (5, 6)]. The activation of cytotoxic NK cells to kill their targets depends on the balance between the aforementioned activating and inhibitory receptors. Upon recognizing their targets, NK cells exert direct cytotoxic activity by releasing cytotoxic granules containing perforin and granzymes or by antibody-dependent cell cytotoxicity (ADCC) (7). Regulatory NK cell activation is induced by exogenous cytokines, such as IL-2 and/or IL-15, secreted by immune cells (dendritic cells, macrophages) and cells from infected tissue (8), leading to their secretion of a broad range of cytokines. One of the major activators of NK cell functions is calcium ions. The entry of extracellular Ca^2+^ in NK cells through Ca^2+^ channels is necessary for optimizing granule exocytosis and cytokine production (9–11).

NK cells are main actors of the skin’s innate immune system. They take part in the first line of defense against infectious agents after cutaneous injury and are recruited, with other immune cells, within the first 24h (12). Immediately upon injury, a wave of calcium ions is detected within the wound bed (13) and will persist for 5 days post-wounding, overlapping with the maximal inflammatory activity (14). The propagation of the Ca^2+^ wave, considered one of the earliest wound signaling events and initiated by ATP released from damaged cells, increases cytoplasmic Ca^2+^ in surrounding healthy cells, hence activating them (15). Activated in the wound, NK cells exert their cytotoxic activity and produce immunomodulatory cytokines, notably IFN-γ and TNF-α (16), which activate macrophages among other immune cells. Activated macrophages participate in wound debridement and are key regulators of wound healing (17, 18).

Aberrant innate immune response and, notably, defects in NK cell activity have been reported in obese, diabetic, and elderly patients who are prone to infection (8–12). These populations are also at high risk of developing chronic wounds, which easily get infected (19). Only a few reports have been published on the involvement of NK cells in the defense and healing processes following cutaneous injury (16, 20, 21). Developing strategies to stimulate NK cell activation may be one way to fight local infection and improve wound healing, especially in these patients whose innate immune response is compromised.

Alginate dressings are made of alginate polymers (extracted from brown algae) composed of guluronic (G) and mannuronic acid (M) units bound with calcium ions. The M/G ratio varies considerably from one alginate dressing to another because it depends on the specie of the algae used, its geographic origin, the harvesting season, the part selected (leaf or stem), etc. (22). The M/G ratio influences Ca^2+^ chelation and its consecutive release from the dressings into the wound (23). Some alginate dressings are supplemented with carboxymethylcellulose (CMC), aiming to increase their absorption capacity. Thus, the performance and biological activity of calcium alginate dressings differ significantly.

This study evaluated the activation of NK cells by exogenous Ca^2+^ released from calcium alginate dressings, widely used for wound treatment. A CaCl_2_ solution was chosen as a third comparator because it contains free Ca^2+^, easily available for cell stimulation. The study focused on the analysis of the cytotoxic capacity and cytokine production of NK cells in contact with two alginate dressings, Algosteril^®^ (ALG, pure calcium alginate) and Biatain^®^ Alginate (BIA, calcium alginate + CMC) and a 3mM CaCl_2_ solution. Furthermore, the molecular mechanisms involved in Ca^2+^ influx into NK cells were characterized for ALG.

## Materials and methods

### Preparation of conditioned medium from calcium alginate wound dressings

Conditioned media (CM) from a pure calcium alginate dressing (ALG, Algosteril^®^, Laboratoires Brothier) and a calcium alginate dressing composed of 85% Ca^2+^ alginate and 15% carboxymethyl cellulose (CMC) (BIA, Biatain^®^ Alginate, Coloplast) were prepared according to the European recommendations of ISO 10-993-1: 1 cm^2^ of dressing was incubated at 37° C during 24 h in 1 ml of culture medium, either Roswell Park Memorial Institute medium RPMI1640, supplemented with 10% fetal bovine serum (FBS), 1% L-glutamine and 1% penicillin-streptomycin (complete R10 medium) or Hanks Balanced Salt Solution (HBSS) without Ca^2+^ and Mg^2+^. To remove residues of wound dressing fibers, CM were filtered twice using 0.45 µm filters (VWR International). Control-CM was prepared by a 24h-lasting incubation of complete R10 medium at 37°C. CM were stored at 4°C for a maximum of one week and used half diluted in the corresponding culture medium in all experiments.

The CM of a pure CMC hydrofiber dressing (AQUACEL Extra^®^, Convatec) was prepared as described in complete R10 medium. It was used diluted by one-half (CMC-CM) or one-fifth in order to get a CMC concentration close to BIA-CM (CMC-CM1/5).

### Preparation of the 3mM CaCl_2_ solution

A 2.4M sterile Ca^2+^-containing solution was prepared by adding calcium chloride (CaCl_2_, Sigma) into deionized water and diluted in complete R10 medium to a final 3mM Ca^2+^ concentration. The 3mM concentration was chosen to be in the range of the concentration of Ca^2+^ released by the studied alginate dressings. At this concentration, the chloride ion supply is negligible compared to the physiological chloride ion concentration (approximately 150mM).

### Concentration of calcium ions in CM and in exudate

The concentration of calcium ions released in control-CM and alginate dressing CM over the 24-hour incubation at 37°C was quantified using inductively coupled plasma/atomic emission spectrometry by an external laboratory, Flandres-Analyses (Capelle la grande, France), according to the NF EN ISO 11885 standard. In addition, the calcium concentration was also measured in wound exudate wastes that are removed by seroma evacuation from the surgical wounds to be discarded by the nurses for wound management.

### Purification of NK cells from PBMCs

Human peripheral blood mononuclear cells (PBMCs) were isolated from buffy coats of healthy donors obtained from blood bank facility EFS (Paris, France) by centrifugation on a Ficoll density gradient (d=1.077 g/mL, lymphocyte separation medium, Eurobio Scientific-France). NK cells were purified from the isolated PBMCs using a negative immunomagnetic selection (Miltenyi Biotec). NK cell purity was assessed by flow cytometry using anti-CD3 and anti-CD56 antibodies. Populations with at least 95% of CD56^+^CD3^-^ isolated cells were considered pure NK cells.

### NK cytotoxic activity assay against K562 target cells

The myelogenous leukemia cell line K562 was maintained in complete R10 medium and used as target cells for NK cells. This streamlined, commonly used method replaced radioactive methods, was amenable to both diagnostic and research applications, and provided information on NK cell cytotoxic activity, whatever the field of study. To discriminate between K562 and NK cells, 5x10^5^ cells/ml K562 cells were stained with a fluorescent dye Carboxy Fluorescein Succinimidyl Ester (CFSE) (Invitrogen) at a concentration of 1 µM for 10 min at 37°C, and then washed twice in complete R10 medium. NK cells (effector cells) were incubated with a fixed number of stained K562 cells, with effector: target (E:T) ratios of 10:1, 5:1, and 1:1 for 18h at 37°C in a humidified incubator under a 5% CO_2_ atmosphere. After incubation, the dye 7-Aminoactinomycin D (7-AAD, Sigma) was added to the cells to quantify the % of K562 dead cells by flow cytometry (BD LSRFortessa). To quantify NK cell cytotoxic activity, the percentage of lysis of K562 cells by NK cells was obtained by the percentage of 7-AAD positive cells among CFSE positive K562 cells. CFSE-unstained K562 cells and CFSE-stained K562 cells, both without NK cells, in the presence or not of 7-AAD addition, were used as non-NK cell containing controls. Data were analyzed with the FlowJo (BDBiosciences) software.

### CD107a degranulation assay

NK cells were mixed with K562 cells at a 1:1 ratio for a co-culture of 18h at 37°C. Thereafter, the cells were spun down and stained for 30 min on ice with fluorochrome-conjugated monoclonal antibodies anti-CD56-APC (BD Biosciences) and anti-CD107a-FITC (BD Biosciences) in staining buffer composed of phosphate-buffered saline (PBS) supplemented with 0.5% of bovine serum albumin (BSA) and 2mM of ethylene diamine tetraacetic acid (EDTA) (Invitrogen). Cells were washed twice with PBS and fixed in 4% paraformaldehyde (PFA). To quantify degranulation by NK cells, the percentage of CD107a-positive cells among CD56^+^ cells was measured by flow cytometry. Data were analyzed with the FlowJo (BD Biosciences) software.

### NK cell phenotyping

Cell surface staining was performed on NK cells after 18h of incubation with Control-CM, ALG-CM, or 3mM CaCl_2_ in R10 complete medium. Cells were stained in staining buffer with fluorochrome-linked monoclonal antibodies: anti-CD14-FITC (BD Biosciences), anti-CD3-FITC (Biolegend), anti-CD56-AF700 (BD Biosciences), anti-CD16-BUV395 (BD Biosciences), anti-CD69-BUV737 (BD Biosciences), anti-DNAM-1-PE-Vio770 (Miltenyi), anti-NKp30- anti-BV421 (BD Biosciences), anti-NKp46-BV786 (BD Biosciences), anti-NKG2D-BV650 (BD Biosciences), anti-KIR2D-PE (Miltenyi), anti-KLRG1-APC-Vio770 (Miltenyi), anti-CD95-APC (Biolegend), anti-CD85j-PE-Cy5 (BD Biosciences), anti-CD158e1/e2 PerCP-Vio700 (Miltenyi), anti-TIGIT-BV605 (Biolegend), and Fixable Viability Dye eFluor 506. The expression of activating or inhibitory receptors was analyzed on NK cell subsets based on CD16 and/or CD56 expression. Samples were analyzed using an LSRFortessa cytometer (BD Biosciences), and results were generated by FlowJo software.

### Cytoplasmic calcium influx assay

NK Cells were loaded with Fura red AM, a calcium indicator dye (2µM; Invitrogen), in HBSS without Ca^2+^ and Mg^2+^ for 30 min at 37°C. NK cells were then washed to remove the extracellular dye and resuspended at 2x10^6^ cells/ml in HBSS. Fura red AM is excited at 406 nm and emits at 670 nm when bound by Ca^2+^ and excited at 488 nm and emits at 670 nm when unbound. The Fura red ratio (fluorescence intensity of bound Ca^2+^/fluorescence intensity of non-bound Ca^2+^) reports changes in intracellular calcium concentration (24).

Fura red AM-stained NK cells were incubated with (HBSS) Control-CM, ALG-CM, 3mM CaCl_2_ solution, or Ionomycin. Control-CM was used as a baseline, and Ionomycin (1µg/ml; Sigma), an ionophore that raises cytoplasmic Ca^2+^ levels independently of activated receptors, was used as a positive control. When indicated, the Ca^2+^-chelator EDTA (10mM) was added to ALG-CM. Intracellular calcium fluorescence intensities (Fura red ratio) were recorded by flow cytometry for 1min 30 sec from the start of incubation.

To characterize the mechanism of Ca^2+^ influx, the calcium channel blockers, Thapsigargin (TG) (Sigma) and 2-Aminoethoxydiphenyl borate (2-APB) (Sigma), were used. Fura red AM-stained NK cells were incubated with either 1µM TG or 100µM 2-APB for 20 min prior to incubation with ALG-CM or 3mM CaCl_2_ solution. Intracellular calcium fluorescence intensities (Fura red ratio) were recorded in the presence of the calcium channel blockers during 1min 30 sec from the start of incubation with ALG-CM or 3mM CaCl_2_ by flow cytometry.

### Intracellular cytokine analysis

NK cells were incubated for 24h with recombinant human IL-2 (10 ng/ml, Peprotech) and IL-15 (100 ng/ml, Peprotech) in complete R10 medium. NK cells were stimulated by either PMA/IONO (150ng/ml of phorbol 12-myristate-13 acetate, PMA, with 3µg/ml ionomycin, IONO) for 4 hours or by lipopolysaccharide LPS (1µg/ml) purified from *Escherichia coli* O55:B5 (Enzo) for 18 hours either in Control-CM, ALG-CM, BIA-CM or 3mM CaCl_2_ solution. Monensin (2µM, BD Biosciences), a protein transport inhibitor, was added to prevent cytokine secretion during the 4 hours of PMA+IONO stimulation or the last 4 hours of LPS stimulation. Cells were then labeled with anti-CD56-APC and anti-CD16-BV711 in the staining buffer for 30 min at 4°C. Cells were washed, fixed, permeabilized using Cytofix/Cytoperm (BD Biosciences), and stained intracellularly with fluorochrome-conjugated monoclonal antibodies anti-IFN-γ-PE and anti-TNF-α-AF488. Finally, cells were washed and analyzed by flow cytometry.

### Statistical analysis

Results were expressed as mean ± SEM from independent experiments. Wilcoxon paired test were performed using Prism (GraphPad 9.1.1): §: p < 0.1; *: p<0.05, **: p<0.01.

## Results

### Calcium level measurement in cutaneous wound exudate samples and conditioned media from alginate dressings

The Ca^2+^ levels within cutaneous lesions have been measured in wound exudate collected during the postoperative period, within the first two weeks after breast reduction mammaplasty (n=6). A mean Ca^2+^ concentration of 2.2 ± 0.3 mM has been detected in wound exudate. The measurements of the concentration of Ca^2+^ released in the conditioned medium (CM) of alginate dressings indicated a mean of 0.59 ± 0.06, 3.6 ± 0.09, and 4.08 ± 0.08 mM for complete R10 medium, ALG-CM, and BIA-CM, respectively. This indicates that calcium levels in wound exudates are in a similar range to Ca^2+^ levels released by alginate dressings in CM.

### Cytotoxic activity of NK cells

To determine whether exogenous calcium stimulates NK-cell cytotoxic activity, cytotoxic activity was assessed after the co-incubation of primary NK and K562 cells during 18h at different effector: target (E:T) ratios.

**Figure 1:**
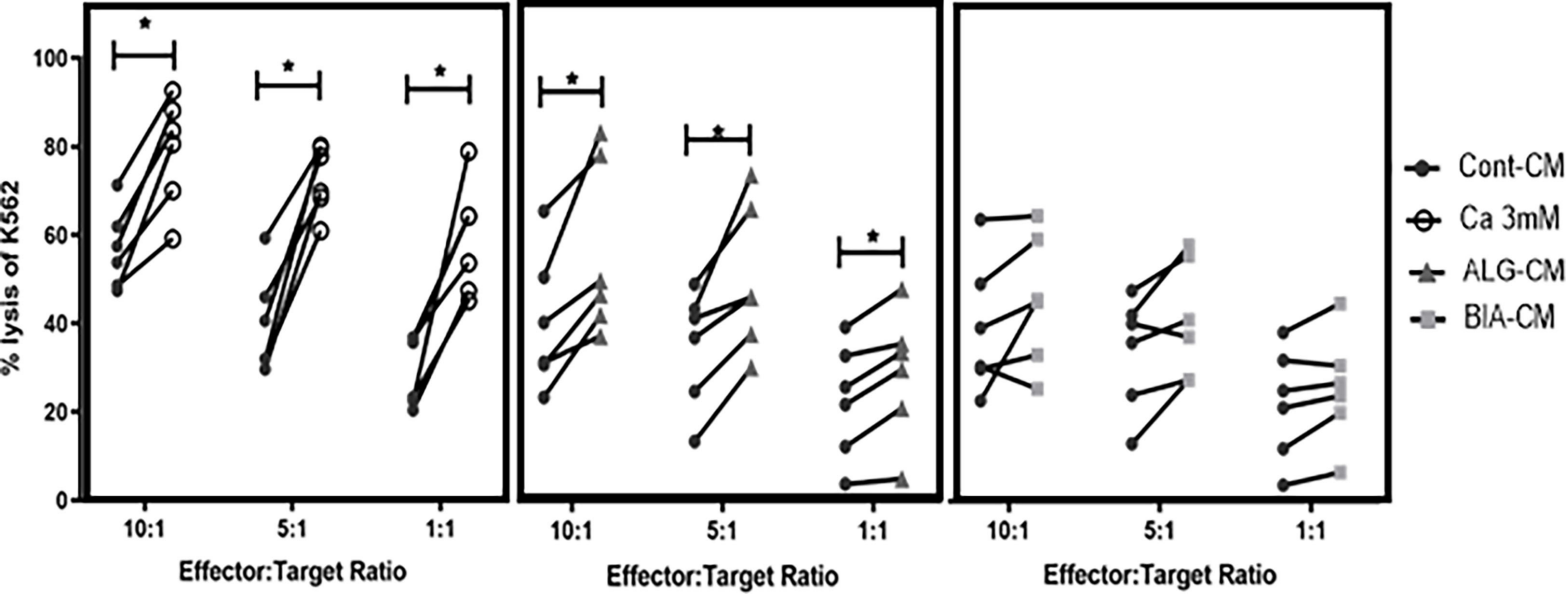
Cytotoxic activity of NK cells. The cytotoxic activity of NK cells was assessed by using purified NK cells from healthy donors as effector cells and K562 cell line as target cells at the indicated effector: target (NK: K562) ratios of 10:1, 5:1, and 1:1. NK cells were mixed with CFSE-labeled K562 for 18h before analysis of CFSE and 7-AAD double positive dead K562 cells by flow cytometry. Cells were incubated with a CaCl_2_ solution at 3 mM (white circle, left side), conditioned medium from either calcium alginate dressing ALG-CM (grey triangle, middle) or BIA-CM (light grey square, right side) *versus* control medium (dark circle). Each line links data obtained from the same donor (n=6); Respective control cytotoxic activity at each ratio was used for statistical comparison using Wilcoxon paired test. *p<0.05.

The 3mM CaCl_2_ solution, used at a concentration close to the one detected in wound exudate, increased the cytotoxic activity of NK cells at all E:T ratios as compared to the respective untreated controls (CT) of each ratio (**Figure 1**, left; **Supplementary Figure 1**). ALG-CM significantly increased the cytotoxic activity of NK cells against K562 cells for all the E:T ratios (**Figure 1**, middle), whereas BIA-CM did not significantly alter NK cell cytotoxic activity (**Figure 1**, right). These results indicate that 3mM calcium and ALG-CM, but not BIA-CM, enhance the capacity of NK cells to kill their targets.

The difference in action of the two calcium alginates could be related to the presence of CMC in BIA-CM and not in the pure ALG-CM. NK-cell cytotoxic activity in the presence of CMC-CM showed a significant decrease in K562 lysis as compared to Cont-CM (**Supplementary Figure 2**). Of interest, the addition of CMC to ALG-CM reduces NK cell cytotoxic activity at the level of Cont-CM and Bia-CM, confirming the negative effect of CMC on calcium alginate action.

### CD107a surface expression on NK cells

**Figure 2:**
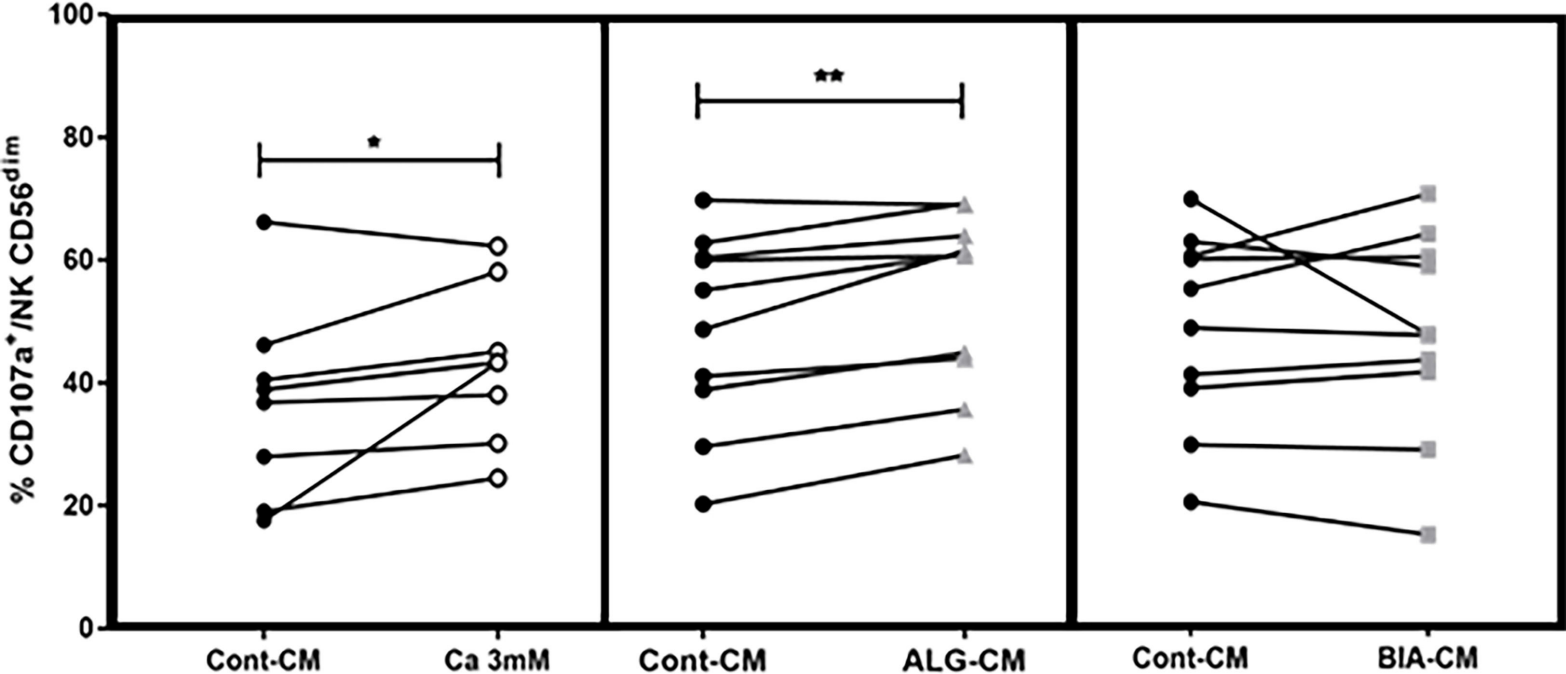
Degranulation of NK cells. The CD107a surface expression was assessed on viable CD56^+dim^ cytotoxic cells by flow cytometry after NK cells incubation with K562 cells at a 1:1 ratio for 18h in the presence of CaCl_2_ solution at 3mM (white circle, left side), ALG-CM (grey triangle, middle), and BIA-CM (light grey square, right side) *versus* control (black circle). Each line links data from the same donor (n=8 for Ca 3mM; n=10 for ALG-CM and BIA-CM). Statistical comparison of data *versus* control CD107a expression was assessed using Wilcoxon paired test. *p<0.05, **p<0.01.

NK cell degranulation following stimulation can be quantified by determining their surface expression of the lysosome-associated membrane protein-1 (LAMP-1/CD107a) (25). Herein, the effects of 3mM CaCl_2_, ALG-CM, or BIA-CM were studied on CD107a expression by NK cells after incubation with K562 at a 1:1 ratio during 18h. Compared to the control medium, CaCl_2_ solution and BIA-CM had no effect on CD107a expression among NK CD56^+^, whereas CD107a expression was significantly increased in the presence of ALG-CM, especially on CD56^dim^ subpopulation, as shown in **Figure 2**. These results demonstrate that the conditioned medium with the pure alginate dressing ALG induces the activation of NK cells by increasing their degranulation. To note, no effect of CMC addition to ALG-CM or control CM was detected on CD107a expression (data not shown).

### Lymphocyte activation marker CD69 on NK cells is up-regulated in the presence of exogenous Ca^2+^ and ALG-CM

**Figure 3:**
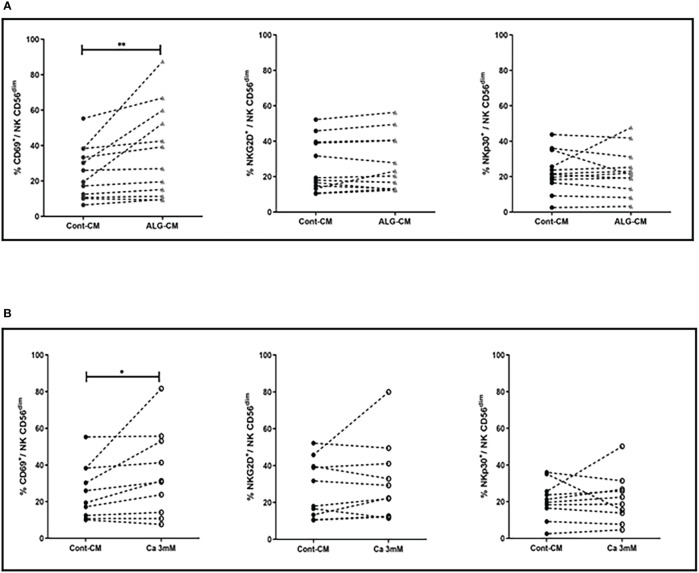
Activating receptor expression on NK cells. Expression levels of NK cell activating receptors: CD69 (left side), NKG2D (middle), and NKp30 (right side) were studied on NK CD56^dim^ after cell incubation for 18h with: **(A)** CaCl_2_ solution at 3mM (white circle), **(B)** ALG-CM (grey triangle). Each line links data from the same donor (A: n=10) and (B: n=12). Statistical comparison of data *versus* control expression (dark circle) was assessed using Wilcoxon paired test *p<0.05, **p<0.01.

Having observed an increase in NK cell-mediated cytotoxic activity in the presence of exogenous CaCl_2_ solution and ALG-CM, the expression of NK cell-activating or inhibitory receptors was further studied on NK cells. Both CD56^dim^ and CD56^bright^ NK cell subsets were analyzed for their membrane phenotype. The main modification observed under exogenous CaCl_2_ solution and ALG-CM was the increase in the expression of the CD69 activator receptor within the CD56^dim^ NK cells, as shown in **Figure 3** (A-B, upper and lower left side). The results did not show any significant changes in the expression of the epitopes NKG2D and NKp30 (**Figure 3**, A-B, upper and lower middle, and right side) or of the other membrane receptors analyzed (data not shown).

### Ca^2+^ influx in NK cells is induced in the presence of exogenous Ca^2+^ and ALG-CM

**Figure 4:**
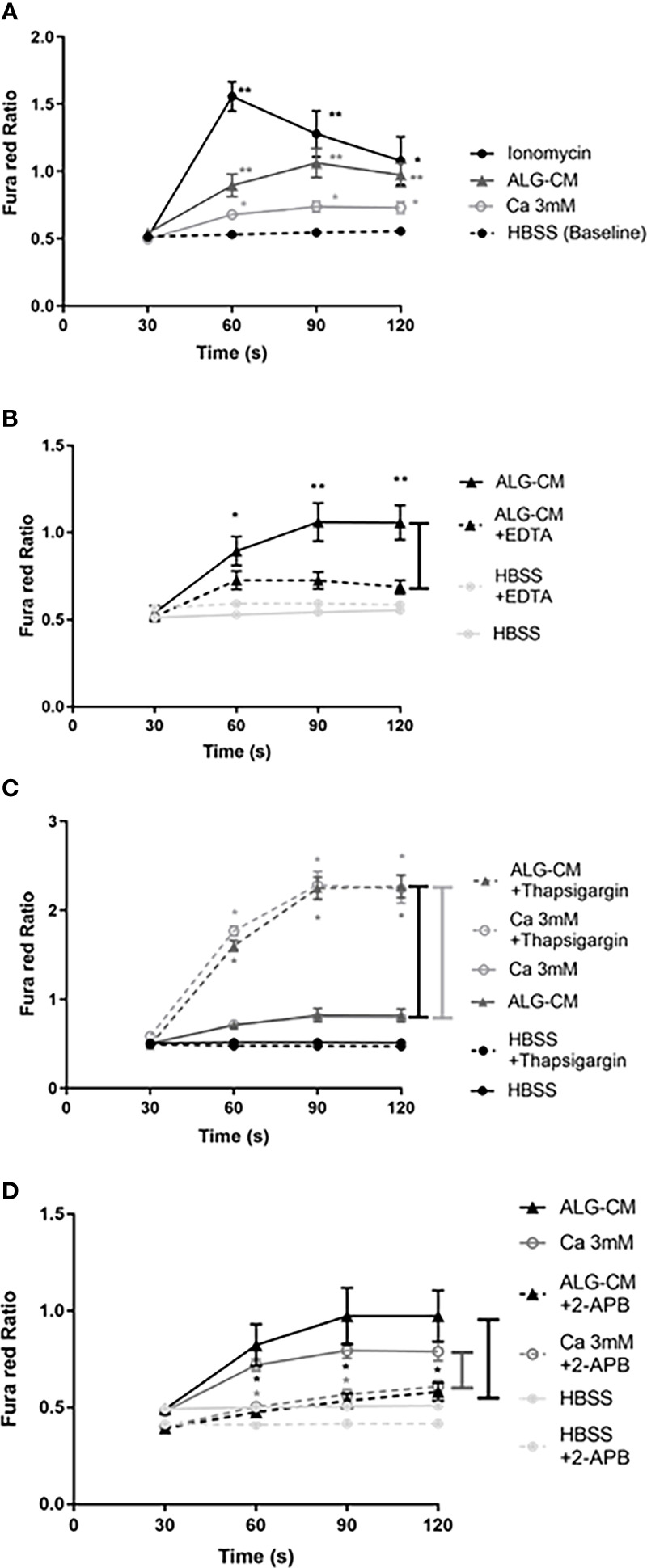
Fura red-AM Cytoplasmic calcium influx in NK cells. Time-dependent calcium influx in NK cells was measured by the Fura red Ratio detected every 30 seconds until 120s in each time interval (30s, 60s, 90s, and 120s) in HBSS solution. Ca^2+^ influx was measured after: **(A)** the addition of ionomycin as a positive control (1µg/ml) (circle, fill line), CaCl_2_ solution at 3mM (white circle), ALG-CM (triangle), compared with control HBSS solution (baseline); **(B)** chelation of extracellular Ca^2+^ released from ALG-CM using EDTA (10mM) diluted in conditioned medium (triangle, detached line) *versus* ALG-CM without EDTA (triangle, full line) or EDTA (10mM) diluted in HBSS (light grey circle, detached line) *versus* HBSS without EDTA (light grey circle, full line); **(C)** calcium store depletion with Thapsigargin (1µM), in the presence of CaCl_2_ solution at 3mM (white circle) or ALG-CM (triangle); **(D)** blockade of Ca^2+^ permeable channels 2-APB (100µM) in the presence of CaCl_2_ solution at 3mM (white circle) or ALG-CM (triangle) or HBSS (light grey circle). Results are the mean ± SEM of independent experiments, using Wilcoxon paired test for statistical comparison to **(A)** HBSS baseline at each time interval with n=9, to **(B)** respective media without EDTA with n=9, to **(C)** respective media without Thapsigargin with n=6 or to **(D)** respective media without 2- APB with n=6. *p<0.05, **p<0.01.

Changes in intracellular Ca^2+^ levels in NK cells following stimulation with ALG-CM and exogenous Ca^2+^ were measured using Fura red-AM, as shown in **Supplementary Figure 3** for one representative experiment. Following the addition of ALG-CM or 3mM CaCl_2_ solution, a significant increase of the Ca^2+^ influx in NK cells was measured over 2 min as compared to baseline (**Figure 4A**). These data highlighted the role played by the calcium released from the pure calcium alginate dressing in inducing the activation of intracellular calcium signaling in NK cells. The calcium alginate BIA was excluded from this analysis due to an aberrant signal after its addition to the cells (data not shown).

Intracellular calcium signaling involves extracellular calcium entry *via* ion channels and calcium release from intracellular stores, leading to modifications in intracellular calcium levels. To confirm the extracellular Ca^2+^ entry induced by ALG-CM in NK cells, Ca^2+^ influx was studied following the chelation of Ca^2+^ released from ALG-CM by adding EDTA to the conditioned medium. This addition significantly decreased the calcium influx as compared to ALG-CM without EDTA (**Figure 4B**).

To empty intracellular calcium stores before adding 3mM CaCl_2_ solution or ALG-CM, intracellular Ca^2+^ stores were mobilized using Thapsigargin (TG). Thapsigargin depletes ER stores through inhibition of the sarco/endoplasmatic Ca^2+^ ATPase and raises cytoplasmic calcium concentration by inhibiting the ability of the cells to pump calcium into the ER (26).This depletion and blockade of intracellular Ca^2+^ stores allowed us to study extracellular Ca^2+^ entry specifically. This led to a significant increase in Ca^2+^ influx in the presence of the CaCl_2_ solution or ALG-CM as compared to the control (**Figure 4C**). Thus, CaCl_2_ solution and ALG-CM do not trigger Ca^2+^ release from intracellular stores, and they do favor extracellular Ca^2+^ entry. These results suggest that NK cell response to the pure calcium alginate ALG entirely depends on the influx of extracellular Ca^2+^ through the involvement of Ca^2+^ permeable plasma membrane channels.

We questioned whether exogenous calcium from Algosteril triggers Ca^2+^ influx into NK cells *via* the store-operated calcium entry (SOCE) channels. We used the inhibitor 2-APB, which blocks STIM/ORAI1 and ORAI2 (SOCE mechanism), preventing the entry of extracellular Ca^2+^ and modulates the Transient Receptor Potential Ion channel (TRP), as previously described (27). Our results showed that 2-APB significantly inhibited the Ca^2+^ influx induced by CaCl_2_ solution or ALG-CM (**Figure 4D**). The results suggest that the entry of the Ca^2+^ liberated from ALG-CM into NK cells occurs *via* the SOCE channels.

### Effect of the chelation of extracellular Ca^2+^ and blockage of STIM/ORAI on NK cells cytotoxic activity induced by ALG-CM

**Figure 5:**
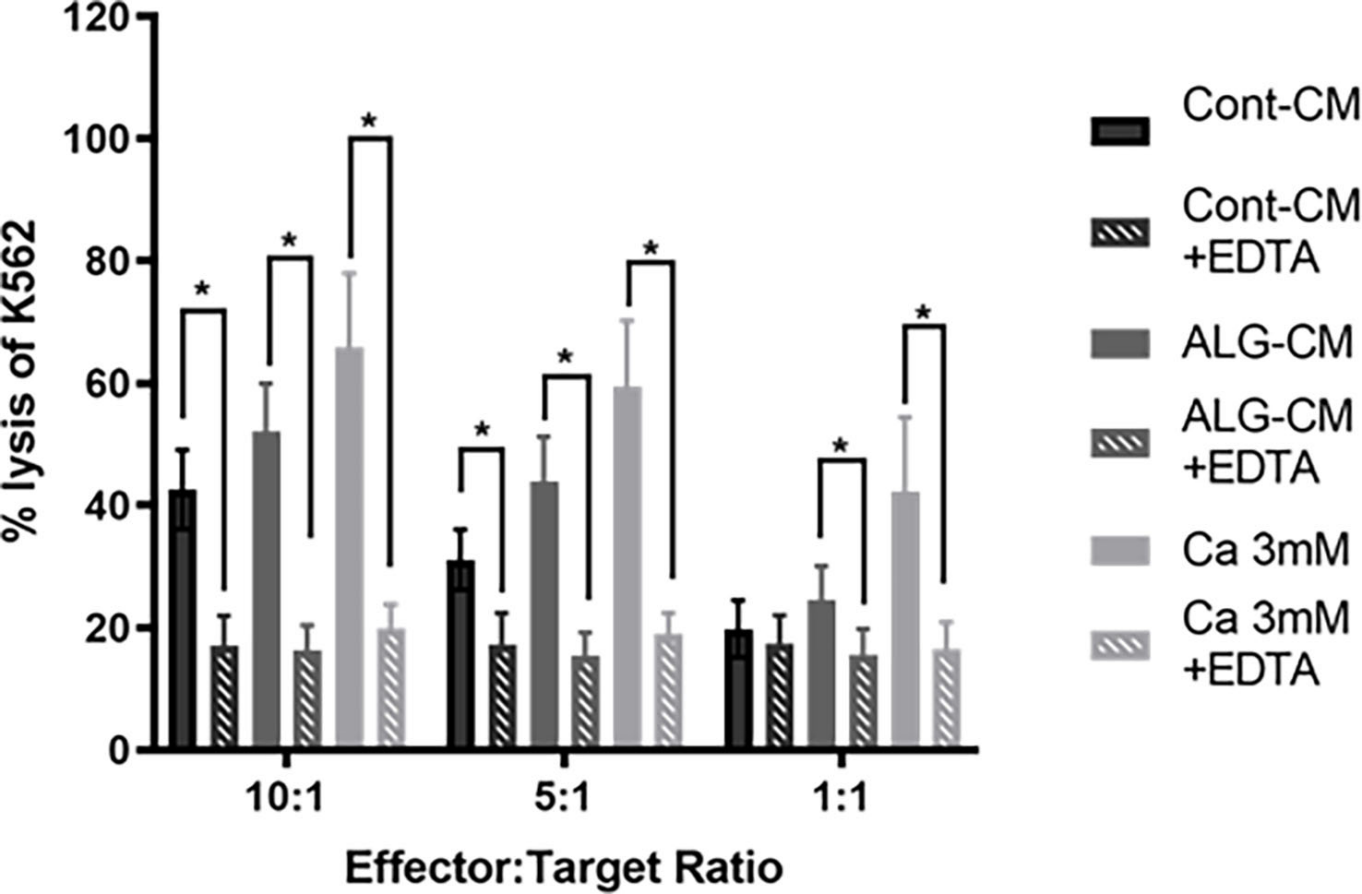
Impact of the depletion of extracellular Ca^2+^ on NK cell cytotoxic activity. The cytotoxic activity of NK cells was assessed in the presence of EDTA by a flow cytometry-based assay using purified NK cells from healthy donors as effector cells and K562 cells as target cells at the indicated effector: target (NK: K562) ratios. EDTA was added to conditioned medium (ALG-CM) (grey hatched bars) or CaCl_2_ solution at 3mM (light grey hatched bars). NK cells were incubated with CFSE-labeled K562 for 18h. Results are the mean of the percentage of 7-AAD positive K562 ± SEM of 6 independent experiments. Statistics compared to each media without the addition of EDTA (filled bars) using Wilcoxon paired test *p<0.05.

Our results suggest that the increased NK cell cytotoxic activity induced by ALG involved Ca^2+^ influx. Thus, we studied the NK cell-mediated killing of K562 cells in the presence or not of 10mM EDTA in either control medium, CaCl_2_ solution, or ALG-CM. The addition of EDTA induced, at E:T ratios of 10:1 and 5:1, significant inhibition of K562 killing by NK cells of respectively 60 and 44.8% in the control medium, 68.8 and 64.9% in ALG-CM and 70 and 68% in CaCl_2_ solution (**Figure 5**). This demonstrates that Algosteril stimulatory effect on NK cell cytotoxic activity is dependent on its exogenous Ca^2+^ release.

### Cytokine production by NK cells cultured with IL-2/IL-15

Another essential function of NK cells upon activation concerns the production of pro-inflammatory cytokines, including IFN-γ and TNF-α. Intracellular cytokine production was induced by stimulation with PMA/IONO or by a TLR dependent-stimulation with LPS.

**Figure 6:**
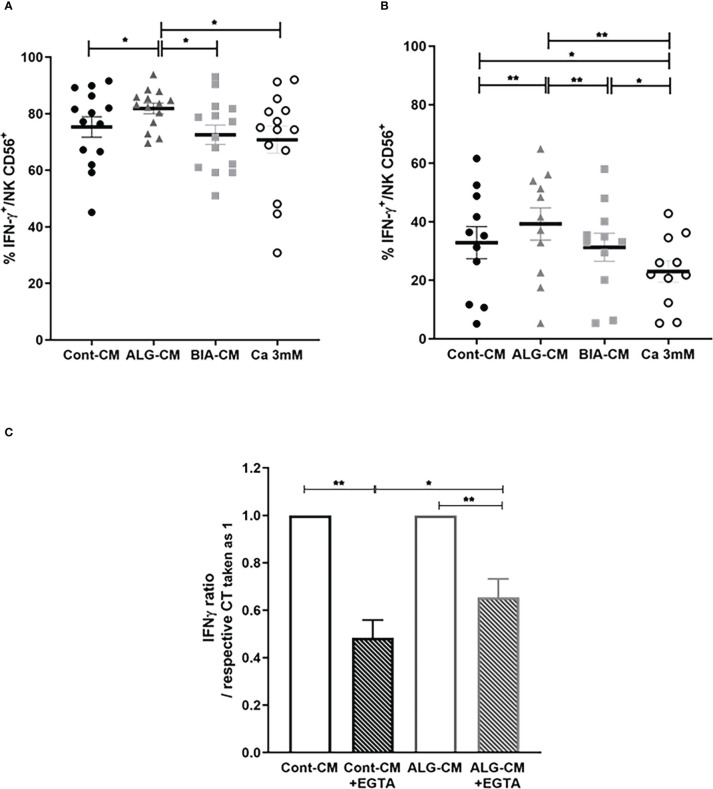
Effects of different sources of exogenous calcium on IFN-γ production by NK cells. Flow cytometric detection of IFN-γ production by purified CD56^+^ NK cells incubated for 24h in complete R10 medium with IL-2 (10ng/ml) plus IL-15 (100ng/ml) then stimulated with **(A)** PMA/IONO for 4h or **(B)** LPS (1µg/ml) for 18h in the presence of ALG-CM (grey triangle), BIA-CM (light grey square), and CaCl_2_ 3mM (white circle) *versus* conditioned control medium (black circle). Results show the % of positive intracellular staining detected on viable CD56^+^ cells of n=14 donors (for PMA/IONO stimulation) and n=11 (for LPS stimulation). **(C)** Ratio of LPS-induced-IFN-γ expression in the presence of EGTA related to the respective controls in the absence of EGTA taken as 1 (white bars). Results are mean ± SEM (n=8), Wilcoxon paired test *p<0.05, **p<0.01.

IFN-γ production by NK CD56^+^ cells induced by stimulation with PMA/IONO was not modified in the presence of CaCl_2_ solution and BIA-CM, while ALG-CM significantly increased IFN-γ production compared to control (**Figure 6A**). Noteworthy, the same results were observed on the regulatory subset NK CD56^bright^ (data not shown). Similarly, the IFN-γ production by NK CD56^+^ cells stimulated with LPS was significantly increased in the presence of ALG-CM, whereas it was not with BIA-CM. It was even significantly reduced with the CaCl_2_ solution (**Figure 6B**). These results suggest a Ca^2+^-independent effect of ALG-CM on NK cell cytokine production, demonstrating a specific activity related to this alginate polymer.

To confirm this alginate polymer-dependent effect on the production of IFN-γ by NK cells stimulated with LPS, the Ca^2+^ was chelated by adding EGTA. The presence of EGTA in the control medium and in ALG-CM reduced the LPS-induced-IFN-γ expression taken as 1 of 0.48 ± 0.21 and 0.26 ± 0.22, respectively (**Figure 6C**). This indicated a lower reduction of IFN-γ production in ALG-CM in the presence of EGTA as compared to the control, clearly showing that the alginate polymer acts on NK cell cytokine production independently of its Ca^2+^ release.

Concerning TNF-α production by NK cells under PMA/IONO stimulation, CaCl_2_ solution tended to decrease its production while ALG-CM tended to increase it (**Supplementary Figure 4A**). Of interest, ALG-CM significantly increased the production of TNF-α in the immuno-regulatory sub-population NK CD56^bright^ in contrast to BIA-CM and calcium solution, which had no significant effect (**Supplementary Figure 4B**). These results confirm that ALG-CM activates NK cell cytokine production through a Ca^2+^-independent mechanism.

## Discussion

NK cells play a key role in the early-activated innate response during wound healing (28) through two main mechanisms: killing of microbes/infected target cells *via* granule exocytosis (29, 30) and regulating the activation of immune cells, notably macrophages, main actors in wound debridement and healing (16–18, 31). Activating NK cells appears as a promising therapeutic strategy to clear pathogens and favor the healing of infected wounds, especially in populations with a compromised innate immune system, such as obese, diabetic, and elderly patients.

Calcium ions are among the most potent activators of fundamental cell functions (32, 33). It was demonstrated that Ca^2+^ is crucial for the activation of NK cell cytotoxic activity and cytokine production (9–11, 34).

The aim of this study was to investigate the activation of NK cells by two calcium alginate dressings known to provide Ca^2+^ to the wound by exchanging their cross-linked calcium ions with sodium ions present in wound exudate (35). The pure calcium alginate Algosteril, the calcium alginate with CMC Biatain Alginate, and a 3mM CaCl_2_ solution were compared. ALG stimulated both NK cell cytotoxic activity and regulatory activity through cytokine production; CaCl_2_ solution only increased NK cell cytotoxic activity, and BIA had no significant effect. Our results further distinguish two NK cell activation mechanisms by ALG, a Ca^2+^-dependent one for NK cell cytotoxic activity and a partially Ca^2+^-independent one for NK cell regulatory activity.

Our results show that ALG and the CaCl_2_ solution increased NK cell cytotoxic activity. To prove that this stimulation is dependent on the Ca^2+^ released in the CM by ALG and the CaCl_2_ solution, a Ca^2+^ chelator was introduced in the medium. This inhibited NK cell cytotoxic activity.

To understand the mechanism involved in the stimulation of NK cell cytotoxic activity by ALG and the CaCl_2_ solution, the Ca^2+^ influx into NK cells was recorded, and a prominent Ca^2+^ influx was triggered. The calcium entry might induce actin reorganization of actin cytoskeleton leading to the release of cytolytic granules containing perforin and granzymes or might also activate signaling pathways such as ERK, NFAT, and NF-κB in NK cells, for gene transcription of cytokines and chemokines, as mentioned in the review published by Paul and Lal. (2017) (36). The introduction of a Ca^2+^ chelator strongly inhibited this influx. The SOCE and ORAI proteins that form Ca^2+^ channels in the plasma membrane (37) enabled this influx, which was demonstrated by blocking them with specific inhibitors. Other types of Ca^2+^ channels like Transient Receptor Potential (TRP) channels, also known to contribute to Ca^2+^ influx in immune cells (27), might be involved.

### NK cell cytokine production: Calcium-independent activation

When NK cells were stimulated with either PMA/IONO or LPS in a pro-inflammatory environment, ALG significantly increased their production of IFN-γ and TNF-α, while BIA had no effect. The CaCl_2_ solution had no effect when NK cells were activated with PMA/IONO and even reduced the production of IFN-γ and TNF-α in the case of LPS stimulation.

The differences in the activation of the NK regulatory activity through cytokine production among the 3 comparators show the involvement of a Ca^2+^-independent mechanism. This Ca^2+^-independent mechanism of ALG was further investigated by adding a Ca^2+^ chelator, which induced a lower reduction of IFN-γ production in the presence of ALG than in the control medium. This demonstrates a partial Ca^2+^-independence of the cytokine production stimulation by ALG. Besides Ca^2+^, the ALG component triggering cytokine production might be linked to the alginate polymer itself: if macrophages recognize alginate through TLR4 binding (which depends on the alginate arrangement of M and G blocks), they produce inflammatory mediators, especially TNF-α (38).

Through the production of cytokines, among which IFN-γ and TNF-α, regulatory NK cells participate in the activation of other immune cell types (39–41), such as monocytes into M1 macrophages (42, 43), neutrophils (44), etc. This activation will boost defense against infection and wound clearing from dead cells, debris, and fibrin. Once wound cleaning is achieved, the inflammatory phase will resolve, which involves the apoptosis of activated immune cells (45, 46). NK cells, *via* their cytotoxic activity, might participate in this apoptotic phase by killing overstimulated macrophages (8, 43) and neutrophils (47). Algosteril, by increasing NK cell cytotoxic and immune-modulatory activities, may thus participate not only in the enhancement of local defense but also in the resolution of the inflammatory phase, thus enabling the activation of the next phase of healing, namely granulation (16, 48, 49).

### Non-equivalence between the two alginate dressings

The two calcium alginate dressings, ALG and BIA, did not induce similar effects on NK cell functions. NK cell cytotoxic activity and cytokine production were enhanced significantly in the presence of ALG, whereas BIA had no effect.

The difference in NK activation by the two alginate dressings could be related to the differences of their chemical composition (M/G ratio), resulting from the manufacturer choice of alginate raw material characteristics (22). The M/G ratio is crucial as it impacts the alginate arrangement of M and G blocks and the release of Ca2+ in the media: the 2 parameters that appear to be involved in NK cell activation in this work. Ca2+ are strongly linked to G blocks compared to M blocks, therefore, the kinetic of Ca2+ release vary according to the M/G ratio.

ALG and BIA media contained more than 3mM Ca2+ (3.6 and 4.1mM respectively). The lack of stimulation of NK cytotoxic activity with BIA could be related to its higher Ca^2+^ concentration as described in the literature: NK cell cytotoxic activity depends on an optimal extracellular Ca^2+^ concentration, lower or higher doses resulting in decreased activation (10). In addition, we demonstrated that CMC, a component of BIA, inhibits NK cell cytotoxic activity, which suggests that CMC might counteract the stimulatory effect of Ca^2+^.

In conclusion, the pure calcium alginate dressing Algosteril is able to stimulate NK cell cytotoxic activity and secretion of cytokines, whereas the CaCl_2_ solution only favors NK cell cytotoxicity. Our data suggest that applying Algosteril on wounds locally stimulates NK cell functions. This stimulation of NK cells should support an efficient inflammatory phase promoting wound defense against pathogens, debris clearance and may accelerate inflammatory phase resolution and healing progression. This would be of particular interest in the case of complex pathological wounds like those observed in patients with an altered innate immune response (obesity, diabetes, and aging). This study proposes a new mode of action for Algosteril, explaining its ability to control infection and accelerate the healing of complex wounds, as proved in clinical trials evaluating Algosteril healing efficacy in the treatment of complex/infected wounds (50–53).

## Data availability statement

The original contributions presented in the study are included in the article/**Supplementary Material**. Further inquiries can be directed to the corresponding author.

## Author contributions

YA carried out the experiments and their analysis and wrote the article. MBo carried out the NK phenotyping for some specific markers. KS, DB, and MM supplied the surgery exudate samples from a previous set of experiments. ND and LM conceptualized NK cell phenotyping studies. CC, LD and MS participated to the collaborative project conception, follow-up and writing of the manuscript. MBa and AB initiated the collaboration between Inserm Unit and Laboratoires Brothier. LM supervised the whole project, including writing, reviewing and editing of the paper. All authors contributed to the article and approved the submitted version.

## Supplementary material

Supplementary Figure 1 NK cytotoxic activity assay. Gating strategy for the analysis of K562 lysis induced by NK cells. (**A**) Dot plots of a representative experiment, using CFSE-unstained K562 and CFSE-stained K562 cells as controls for this experiment (Without NK cells or “non-NK cell containing controls” as called by the reviewer”). (**B–C**) Dot plots of a representative experiment, using K562 cells stained with CFSE before incubation with NK cells and FACS analysis. The lysis of K562, gated as CFSE+ cells, was determined by flow cytometry after 7-AAD staining. Representative histograms of NK cell cytotoxicity towards the K562 cell target at the highest E:T ratio (10:1) are represented as the percentage of 7AAD positive cells among K562 CFSE+ cells in the presence of Cont-CM (black) in (**B**), and in the presence of ALG-CM (dark grey), BIA-CM (grey) and CaCl2 solution (light grey) in lowest histograms (**C**).

Supplementary Figure 2 Cytotoxic activity of NK cells in the presence of a CMC fiber dressing. The cytotoxic activity of NK cells was assessed by using purified NK cells from healthy donors as effector cells and K562 cell line as target cells at 10:1 effector: target (NK: K562) ratio. Cells were incubated with ALG-CM (dark grey bar), BIA-CM (light grey bar), half diluted CMC-CM (white bar), CMC-CM diluted to one fifth (hatched dark greybar), and CMC-CM diluted to one fifth in ALG-CM (hatched light grey bar). Results are the mean ± SEM (*n=7*) of the percentage of K562 lysis. Wilcoxon paired t test, *: *p*<0.05.

Supplementary Figure 3 Fura red-AM Cytoplasmic calcium influx in NK cells. NK cells were loaded with the Ca2+ sensitive dye Fura red-AM. Gated NK cells (left panel FSC-A x SSC-A) on singlets (panel FSC-A x FSC-H) were analyzed by:

$$\text{the ratio}=\frac{\text{bound }{\text{Ca}}^{2+}\text{ fluorescence intensity plotted in the right panels}}{\text{non-bound }{\text{Ca}}^{2+}\text{ fluorescence intensity plotted to the external right panels}}$$

Supplementary Figure 4 Effects of different sources of exogenous calcium on TNF-α production by NK cells. Flow cytometric detection of TNF-α intracellular production by purified total NK CD56^+^ cells **(A)** or NK CD56 ^bright^ **(B)** following 4h with PMA/IONO in the presence of ALG-CM (grey triangle), BIA-CM (light grey square), and CaCl_2_ 3mM (white circle) *versus* conditioned control medium (black circle). Results are the mean ± SEM (n=10). Statistics using Wilcoxon paired test *: p<0.05, **: p<0.01. § tendency p<0.1.
