# Supplementary figures and images for: Modulation of NK cell activation by exogenous calcium from alginate dressings in vitro
Source: Front Immunol. 2023 Apr 6;14:1141047. doi: 10.3389/fimmu.2023.1141047 (PMC10117844; doi:10.3389/fimmu.2023.1141047)

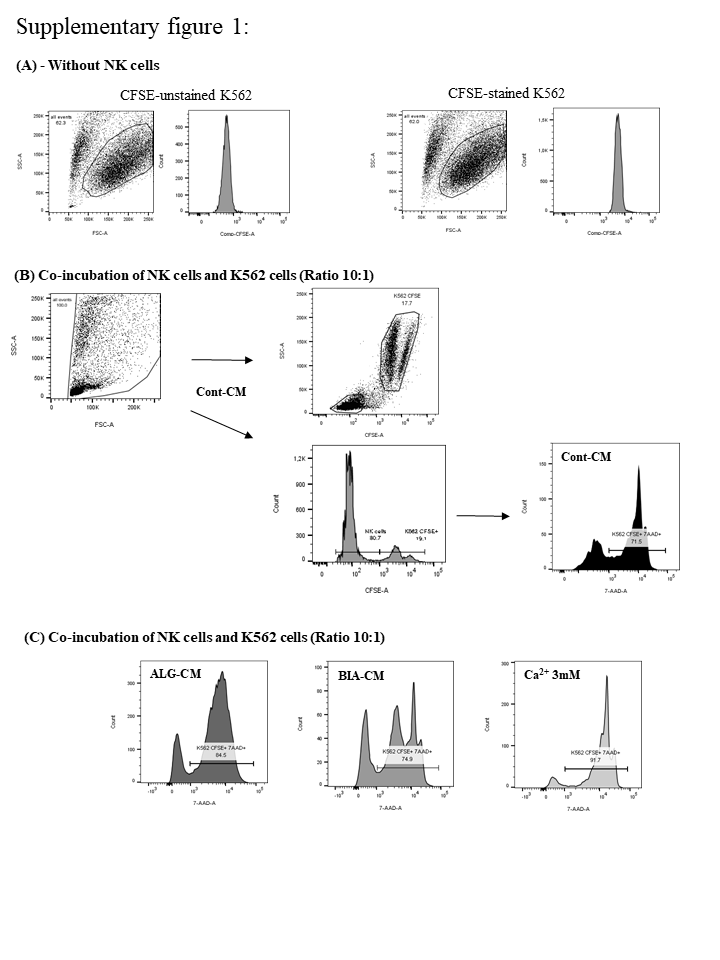

Supplement: Supplementary Figure 1 — NK cytotoxic activity assay. Gating strategy for the analysis of K562 lysis induced by NK cells. (A) Dot plots of a representative experiment, using CFSE-unstained K562 and CFSE-stained K562 cells as controls for this experiment (Without NK cells or “non-NK cell containing controls” as called by the reviewer”). (B–C) Dot plots of a representative experiment, using K562 cells stained with CFSE before incubation with NK cells and FACS analysis. The lysis of K562, gated as CFSE+ cells, was determined by flow cytometry after 7-AAD staining. Representative histograms of NK cell cytotoxicity towards the K562 cell target at the highest E:T ratio (10:1) are represented as the percentage of 7AAD positive cells among K562 CFSE+ cells in the presence of Cont-CM (black) in (B), and in the presence of ALG-CM (dark grey), BIA-CM (grey) and CaCl2 solution (light grey) in lowest histograms (C). [file Image_1.tif]

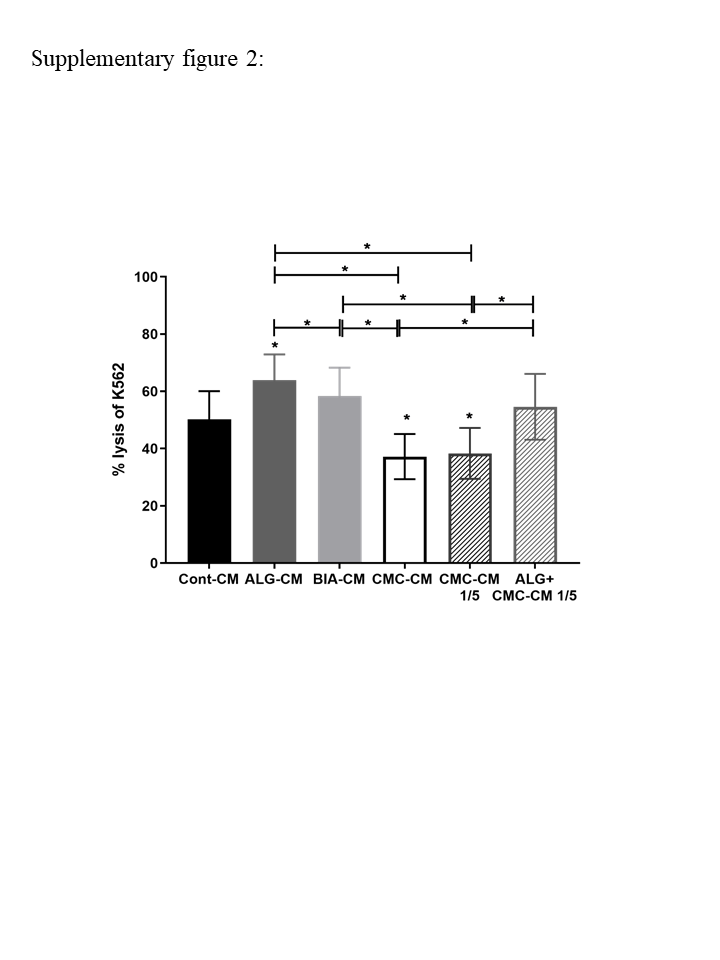

Supplement: Supplementary Figure 2 — Cytotoxic activity of NK cells in the presence of a CMC fiber dressing. The cytotoxic activity of NK cells was assessed by using purified NK cells from healthy donors as effector cells and K562 cell line as target cells at 10:1 effector: target (NK: K562) ratio. Cells were incubated with ALG-CM (dark grey bar), BIA-CM (light grey bar), half diluted CMC-CM (white bar), CMC-CM diluted to one fifth (hatched dark greybar), and CMC-CM diluted to one fifth in ALG-CM (hatched light grey bar). Results are the mean ± SEM (n=7) of the percentage of K562 lysis. Wilcoxon paired t test, *: p<0.05. [file Image_2.tif]

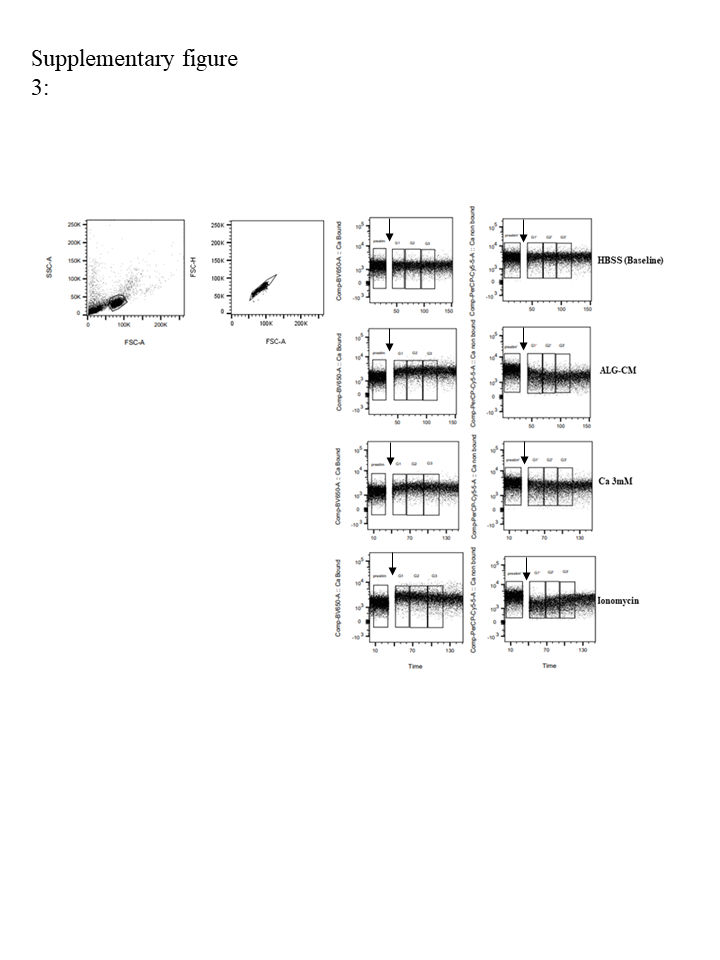

Supplement: Supplementary Figure 3 — Fura red-AM Cytoplasmic calcium influx in NK cells. NK cells were loaded with the Ca2+ sensitive dye Fura red-AM. Gated NK cells (left panel FSC-A x SSC-A) on singlets (panel FSC-A x FSC-H) were analyzed by: [file Image_3.tif]

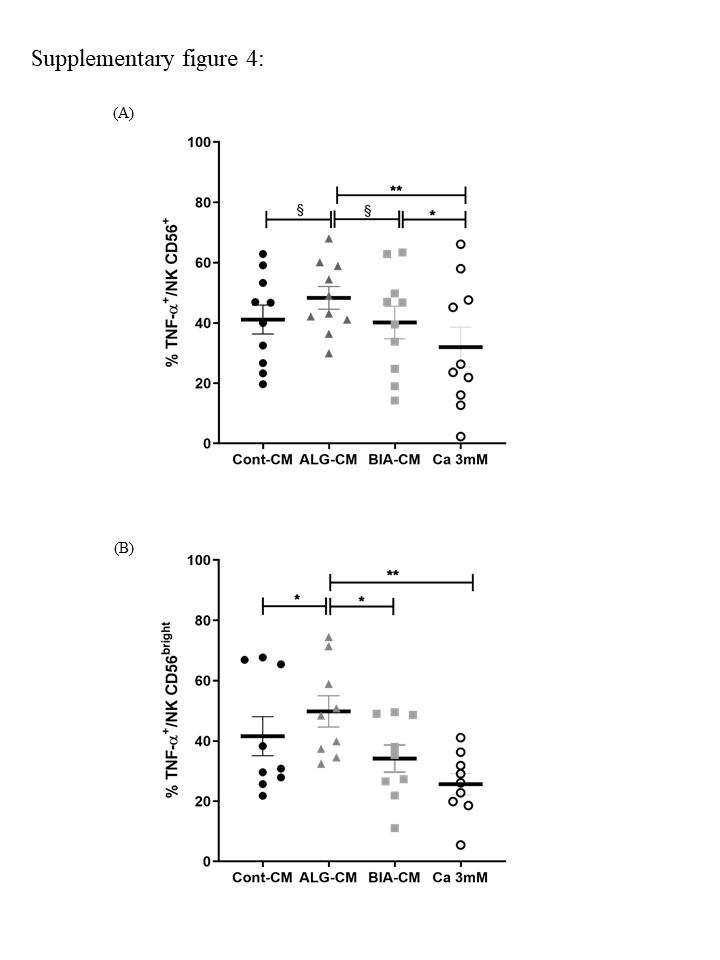

Supplement: Supplementary Figure 4 — Effects of different sources of exogenous calcium on TNF-α production by NK cells. Flow cytometric detection of TNF-α intracellular production by purified total NK CD56+ cells (A) or NK CD56 bright (B) following 4h with PMA/IONO in the presence of ALG-CM (grey triangle), BIA-CM (light grey square), and CaCl2 3mM (white circle) versus conditioned control medium (black circle). Results are the mean ± SEM (n=10). Statistics using Wilcoxon paired test *: p<0.05, **: p<0.01. § tendency p<0.1. [file Image_4.tif]
